# Characterization of key spike RBD residues influencing SARS-CoV-2 variant adaptation to avian ACE2

**DOI:** 10.3389/fcimb.2025.1631926

**Published:** 2025-07-29

**Authors:** Weitong Yao, Yujun Li, Huize Sun, Danting Ma, Xiaojuan Tang, Aiping Zeng, Fang Huang

**Affiliations:** ^1^ Hubei JiangXia Laboratory, Wuhan, Hubei, China; ^2^ Biosafety Level 3 Laboratory of Shenzhen University, Shenzhen, Guangdong, China; ^3^ National Health Commission (NHC) Key Laboratory of Hormones and Development, Tianjin Key Laboratory of Metabolic Diseases, Chu Hsien-I Memorial Hospital and Tianjin Institute of Endocrinology, Tianjin Medical University, Tianjin, China; ^4^ School of Chemical Biology and Biotechnology, Peking University Shenzhen Graduate School, Shenzhen, Guangdong, China; ^5^ Shenzhen Bay Laboratory, Shenzhen, Guangdong, China; ^6^ Department of Ophthalmology, Union Hospital, Tongji Medical College, Huazhong University of Science and Technology, Wuhan, Hubei, China

**Keywords:** SARS-CoV-2, RBD, ACE2, receptor, host range, avian

## Abstract

**Introduction:**

The beta-coronavirus SARS-CoV-2 has been revealed to infect mammals and other species, which potentially promotes the virus adaptation to broader species and the emergence of new variants. The host range of different SARS-CoV-2 variants are mainly determined by the affinity of the receptor-binding domain (RBD) of the spike protein to the host receptor angiotensin-converting enzyme 2 (ACE2). Thus, this study aims to elucidate the detailed mechanisms of such dynamic adaptation of indicated SARS-CoV-2 variants.

**Methods:**

In this study, flow cytometry and surface plasmon resonance (SPR) assays were used to assess the binding affinity between RBDs and avian ACE2. Then, infection assays with MLV-based SARS-CoV-2 spike pseudovirus or authentic viruses were performed to verify the avian ACE2 mediated viral entry. Finally, mutagenesis studies were conducted to identify key amino acids of avian ACE2 orthologs and RBDs.

**Results:**

Our previous findings revealed that wild-type SARS-CoV-2 RBD does not bind chicken ACE2. Here, we found that ACE2 orthologs from chicken and mallard were capable to support binding to RBDs of the Alpha, Beta, and Gamma variants, which further enabled the viral entry. On the contrary, the RBD of BA.1 failed to bind avian ACE2. Whereas, a triple-residue reversal mutant (S446G, S496G, H505Y) restored ACE2 binding and enabled efficient viral entry. Additionally, several key residues within RBD were characterized as the determinant of its affinity to avian ACE2.

**Discussion:**

Our findings reveal that higher mutation rates in emerging variants might lead to future cross-species receptor usage or even spillover. Understanding such cross-species transmission mechanisms provides new insights to the virological features and potential host range of emerging SARS-CoV-2 variants.

## Introduction

Severe acute respiratory syndrome coronavirus 2 (SARS-CoV-2), the etiological agent of coronavirus disease 2019 (COVID-19), has caused a global pandemic since its emergence in late 2019 and the subsequent declaration of a public health emergency in March 2020. SARS-CoV-2 belongs to the genus *Betacoronavirus*, which also includes SARS-CoV and MERS-CoV (Cui et al., 2019; Zhou et al., 2020). Unlike *Gammacoronaviruses* and *Deltacoronaviruses*, which primarily infect birds, *Betacoronavirus* species are traditionally thought to infect only mammals (Cui et al., 2019). However, it has been demonstrated that SARS‑CoV‑2 possesses an unusual capacity for cross‑species transmission, such as outbreaks in farmed mink, and spillback into white‑tailed deer populations. These examples illustrate that the virus can adapt rapidly when introduced into new mammalian reservoirs (Oude Munnink et al., 2021; Palmer et al., 2021). Such cross-species transmission might lead to virus adaptation and the emergence of new variants. Therefore, it is necessary to monitor livestock species for virus spillover.

Receptor usage is generally considered as the determinant of the host range of coronaviruses (Cui et al., 2019). The spike (S) protein displayed on the surface of SARS-CoV-2 mediates the entry to host cells, through the specific and high-affinity binding to host angiotensin-converting enzyme 2 (ACE2) receptor (Jackson et al., 2022). Viral entry begins when the receptor binding domain (RBD) adopts an ‘up’ conformation to dock onto its obligate host receptor, ACE2, via a network of hydrogen bonds and hydrophobic contacts that confer both specificity and nanomolar-range affinity (Wrapp et al., 2020). Following receptor engagement, host proteases (e.g., TMPRSS2) cleave the spike at the S1/S2 and S2 sites, triggering a large-scale conformational rearrangement of the S2 subunit (Hoffmann et al., 2020). Thus, the combined requirements of high-affinity ACE2 binding and compatible proteolytic activation define a two-step molecular “checkpoint” that largely restricts SARS-CoV-2 infection to cells and species.

We and others have found that ACE2 orthologs from a wide range of domestic and wild mammals support SARS-CoV-2 infection (Li et al., 2020; Shi et al., 2020; Liu et al., 2021; Oude Munnink et al., 2021; Hale et al., 2022). Comparative binding and mutational analyses reveal that key RBD substitutions, such as N501Y, E484K, and K417N/T, enhance affinity for certain non-human ACE2 orthologs. The evolution of spike proteins continues to shape their binding affinities to other ACE2 orthologs, facilitating both zoonotic and reverse zoonotic transmission events (Yao et al., 2023). Consistent with this, our previous studies on SARS-CoV and Bat-CoV RaTG13 (SARS-CoV-2-like CoV of pangolin origin) showed that neither of them could utilize chicken ACE2 for cell entry (Li et al., 2020). Consistent with these *in vitro* observations, an *in vivo* infection study showed that turkeys, ducks, chickens, and chicken embryos were found not susceptible to the prototype SARS-CoV-2 (Shi et al., 2020; Berhane et al., 2021). However, a previous *in vitro* study showed that SARS-CoV could use chicken ACE2 (Wang Q. et al., 2021), and another *in vivo* study represented that SARS-CoV did not develop pathologic changes in chicken, while viral RNA was detected in blood and organs at 2 weeks after inoculation (Weingartl et al., 2004). Intriguingly, it has been reported that mutations T478I and N501Y on spike RBD proteins expand the accessibility of SARS-CoV-2 to birds (Wang et al., 2023), hinting that a small number of changes may suffice to overcome the mammal-only host restriction.

To continuously monitor SARS-CoV-2 variants and their host adaptation, indicated binding and infection assays were performed to investigate whether the emerging SARS-CoV-2 variant spikes could bind some avian ACE2 orthologs or utilize them for cell entry. The results showed that avian ACE2 was capable of supporting the entry of the Beta and Gamma variants, as well as emerging Omicron sublineages.

## Results

### Alpha, Beta, and Gamma variants can bind chicken ACE2 and utilize it for entry

**Figure 1:**
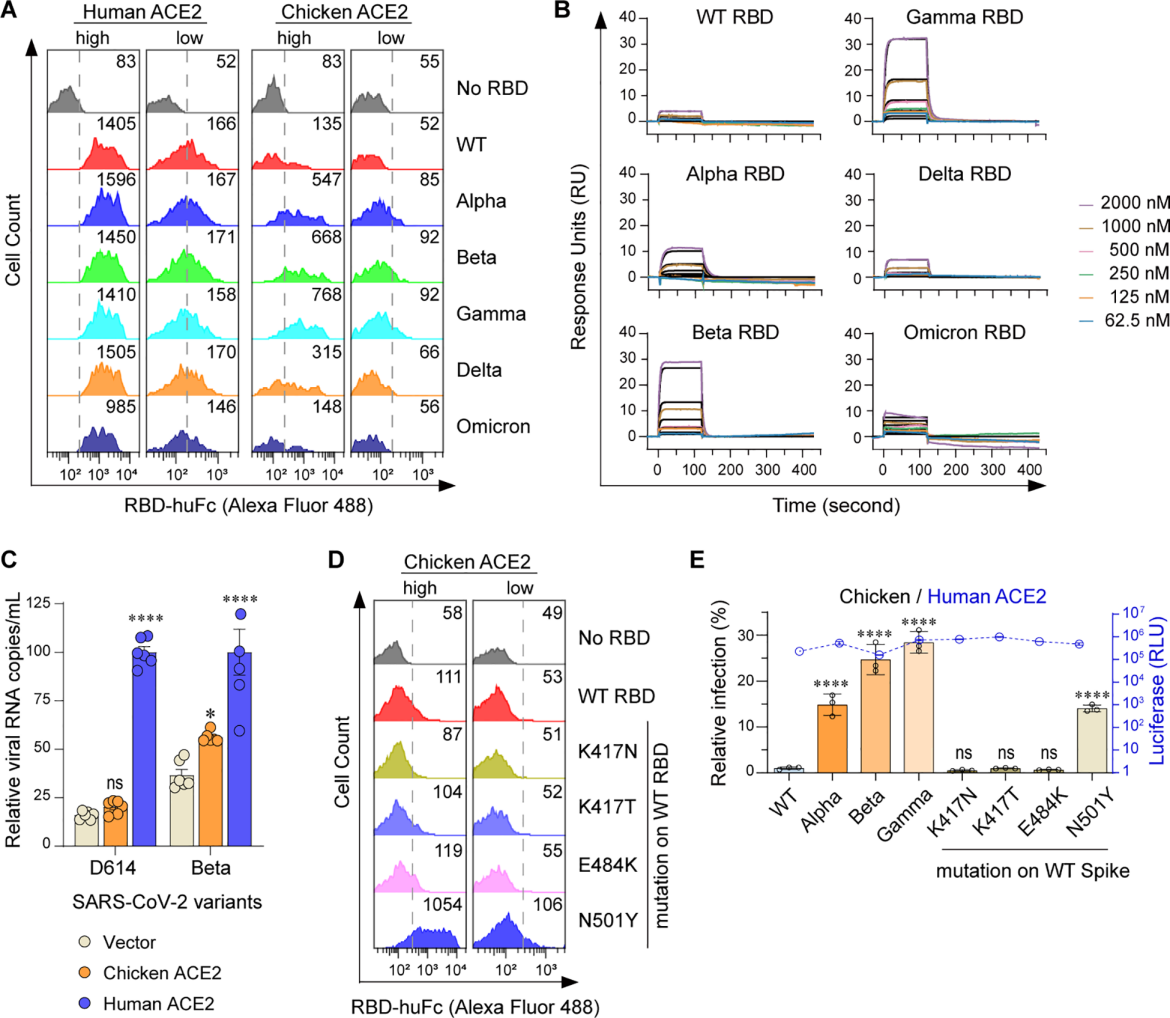
Alpha, Beta, and Gamma variants can bind chicken ACE2 and utilize it for entry. **(A)** Flow cytometry histogram data showing binding of the indicated RBD variants to cell surface-expressed human or chicken ACE2. Based on the intensity of S-tag, cells were gated into two populations, ACE2-high (MFI: 2.5 × 10^3^ to 2.5 × 10^4^) and ACE2-low (MFI: 2.5 × 10^2^ to 2.5 × 10^3^). Numbers in the figure indicate the MFI of RBD-positive cells. **(B)** SPR measurements of interaction kinetics between chicken ACE2 and the indicated monomeric RBD proteins. The raw curves are presented in color; fitted curves are shown in black. **(C)** HeLa cells expressing human and chicken ACE2 were infected with the indicated SARS-CoV-2 authentic virus at an MOI of 0.05 and harvested at 48 hpi for viral RNA quantification. **(D)** Flow cytometry detection of interactions between the indicated RBDs and cell surface expressed chicken ACE2 proteins. **(E)** 293T cells expressing human or avian ACE2s were infected with the indicated SARS-CoV-2 spike-pseudotyped reporter viruses (X-axis). Infection signals of each pseudovirus supported by chicken (bar graphs) ACE2 were calculated as the percentage of infection relative to infection signals of corresponding pseudoviruses supported by human ACE2. Original luciferase signals from human ACE2-supported infection (blue curve) are plotted on the right Y axis. Data are representative of two or three independent experiments and data points represent mean + s.d. ofthree biological replicates. *, *P* < 0.05; ****, *P* < 0.001; n.s., not significant [*P* > 0.05].

We have previously revealed that chicken ACE2 failed to support the cell entry of a prototype SARS-CoV-2 (indicated as WT here) (Li et al., 2020). To investigate whether other SARS-CoV-2 variants interact with chicken ACE2, Alpha (B.1.1.7) (Davies et al., 2021; Volz et al., 2021), Beta (B.1.351) (Tegally et al., 2021), Gamma (P.1) (Diseases JNIoI, 2021), Delta (B.1.617.2) (Dhar et al., 2021; Elliott et al., 2021), and Omicron BA.1 (B.1.1.529) (Cao et al., 2022; Viana et al., 2022) variants were tested. Purified RBD-huFc fusion proteins were used to perform surface staining of 293T cells transfected with plasmids encoding N-terminally S-tagged chicken or human ACE2, followed by flow cytometry analysis. Consistent with our previous findings (Li et al., 2020), chicken ACE2 did not bind to RBDs of WT, Delta, or Omicron variants. Unexpectedly, weak but evident chicken ACE2/RBD binding signals were measured among the Alpha, Beta, and Gamma RBDs (**Figure 1A**; **Supplementary Figure S1A**). To validate the specificity of these interactions, we performed surface plasmon resonance (SPR) assays using a recombinant form of the chicken ACE2 extracellular domain as an immobilized receptor and monomeric RBDs as analytes (**Supplementary Figure S1B**). Indeed, weak but evident interactions were detected between Alpha, Beta, and Gamma RBDs and chicken ACE2 (**Figure 1B**; **Supplementary Table S1**). To test whether these interactions might have biological significance, we evaluated the ability of chicken ACE2 to support SARS-CoV-2 entry. HeLa cells were transfected with human or chicken ACE2 and infected with the SARS-CoV-2 D614 variant or Beta variant. Consistently, chicken ACE2 supported Beta infection but not the D614 virus (**Figure 1C**). In addition, mutations of RBD from Alpha, Beta, and Gamma were individually introduced into the WT strain to test for determinant binding sites. We found that the N501Y mutation in the RBD is the major determinant for gained affinity to chicken ACE2 (**Figure 1D**; **Supplementary Figure S1C**). An MLV-based packaging system enveloped with SARS-CoV-2 spike and incorporating a Gaussia luciferase reporter gene was used to generate pseudoviruses. Chicken ACE2 supported infection of Alpha, Beta, and Gamma variants, as well as pseudoviruses consisting of spikes with the N501Y mutation. Consistent with the binding data, chicken ACE2 supported infection of the Alpha, Beta, Gamma, and N501Y pseudoviruses (**Figure 1E**; **Supplementary Figure S1D**). Our results indicate that the acquired accessibility of SARS-CoV-2 variants (Alpha, Beta, and Gamma) to chicken ACE2 mainly depends on the N501Y mutation in the SARS-CoV-2 RBD.

### Beta and Gamma variants can bind avian ACE2 and utilize it for entry

**Figure 2:**
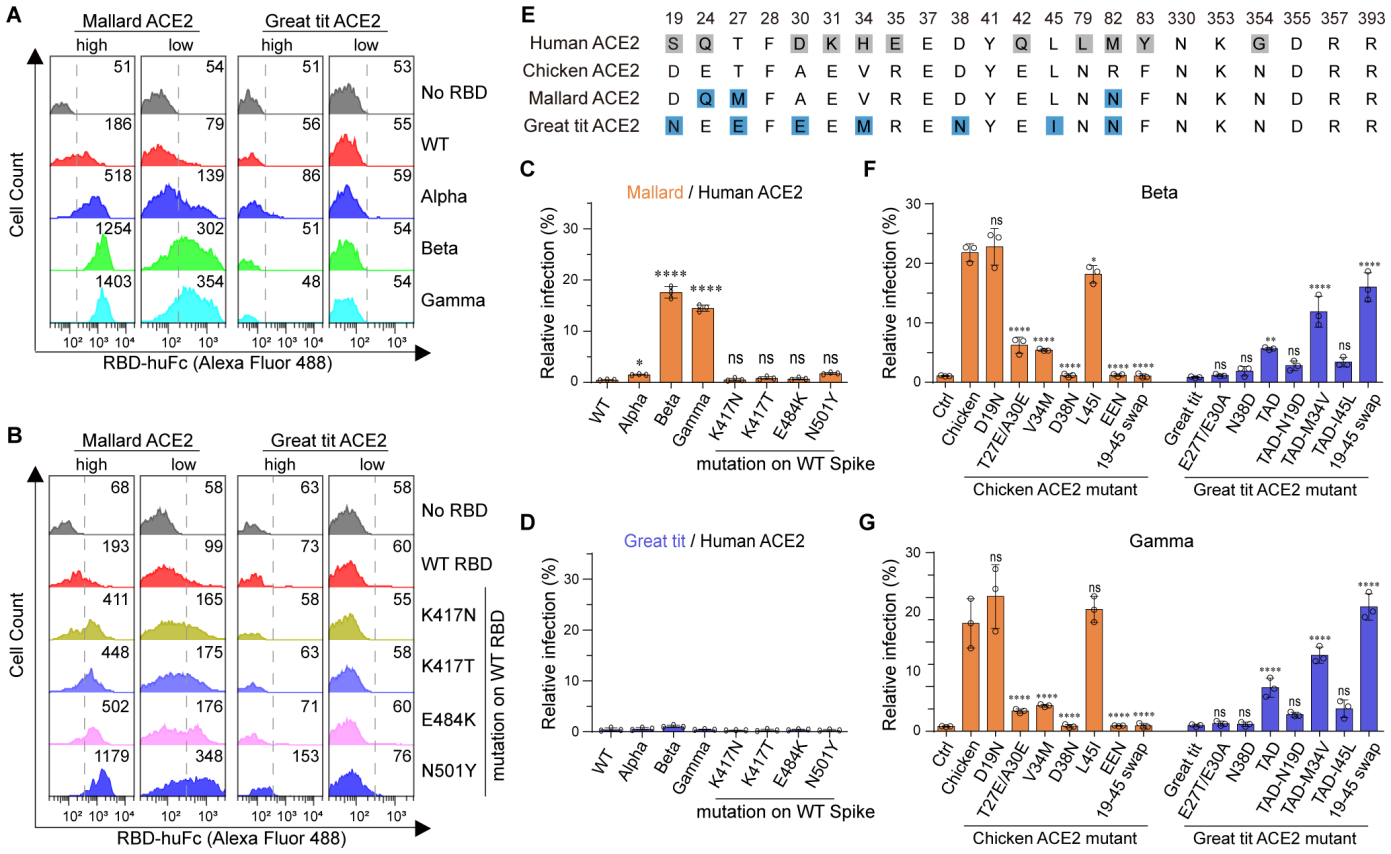
Beta, and Gamma variants can bind avian ACE2 and utilize it for entry. **(A, B)** Flow cytometry histogram data showing binding of the indicated RBD variants to cell surface-expressed mallard or great tit ACE2. **(C, D)** 293T cells expressing the indicated ACE2 were infected with SARS-CoV-2 spike-pseudotyped reporter viruses, similar to **Figure 1E**. Infection signals of each pseudovirus supported by indicated ACE2 proteins were calculated as the percentage of infection relative to infection signals of corresponding pseudoviruses supported by human ACE2. **(E)** Sequence alignment of ACE2 from human, chicken, mallard and great tit. Numbers correspond human ACE2 residues. Gray squares indicate amino acid differences compared to the chicken ACE2 sequence. **(F, G)** 293T cells expressing ACE2 were infected with Beta and Gamma SARS-CoV-2 spike-pseudotyped reporter viruses. Infection signals of each pseudovirus supported by indicated ACE2 proteins were calculated as a percentage of infection relative to infection signals of corresponding pseudovirus supported by human ACE2 (EEN: T27E-A30E-D38N, TAD: E27T-E30A-N38D). ***, *P* < 0.001; ***, *P* < 0.001; **, *P* <0.01; *, *P* <0.05; n.s., not significant [*P* > 0.05].

To further explore the generality of the above findings, we performed amino acid sequence alignment of thirty-one (uniport ~850 ACE2 sequences) randomly selected avian ACE2 orthologs (**Supplementary Figure S2**; **Supplementary Table S2**). The mallard (relatively adaptable to human activities and relevant to influenza transmission) and the great tit (a garden bird with a large population) were selected for the following study. Mallard ACE2 is closely related to chicken ACE2 based on sequence alignment, whereas great tit ACE2 shows substantial differences from chicken ACE2. Interestingly, mallard, but not great tit, ACE2 supported binding to Alpha, Beta, and Gamma RBDs in both ACE2-high and ACE2-low cells (**Figure 2A**; **Supplementary Figure S3A**). We next examined the binding of SARS-CoV-2 variant RBD mutations to these two avian ACE2 orthologs using flow cytometry. The K417N, K417T, E484K, and N501Y mutations contributed to the gained binding affinity to mallard ACE2 in ACE2-high and ACE2-low cells. The N501Y mutation also showed weak binding to great tit ACE2 in ACE2-high cells (**Figure 2B**; **Supplementary Figure S3B**). We then measured the infectivity of SARS-CoV-2 pseudovirus variants in the presence of mallard or great tit ACE2. Although mallard ACE2 binds to all three tested RBDs, it supported infection only by Beta and Gamma pseudoviruses. Great tit ACE2 supported infection by none of these pseudoviruses (**Figures 2C, D**; **Supplementary Figure S1D**). These data demonstrate that mallard ACE2 has the potential to serve as a cellular receptor for Beta and Gamma variants. To further investigate the determinant amino acids of avian ACE2 orthologs that function as entry receptors for SARS-CoV-2 variants, we performed sequence alignment of residues 19–45 among chicken, mallard, and great tit ACE2 proteins (**Figure 2E**). The following mutagenesis studies showed that the D38N mutation completely abolished chicken ACE2-supported infection by Beta and Gamma, while swapping the 19–45 region of chicken ACE2 into great tit ACE2 restored receptor functionality (**Figures 2F, G**; **Supplementary Figures S3C-E**). A single amino acid mutation at chicken ACE2—replacing its D38 residue with the N38 residue from great tit—completely abolished chicken ACE2’s activity in supporting pseudovirus infection, indicating that this residue is essential (**Figures 2F, G**). Taken together, beyond chicken ACE2, mallard ACE2 is also able to mediate the entry of SARS-CoV-2 Beta and Gamma variants.

### Omicron BA.1 RBD reversion mutations enable efficient avian ACE2 binding

**Figure 3:**
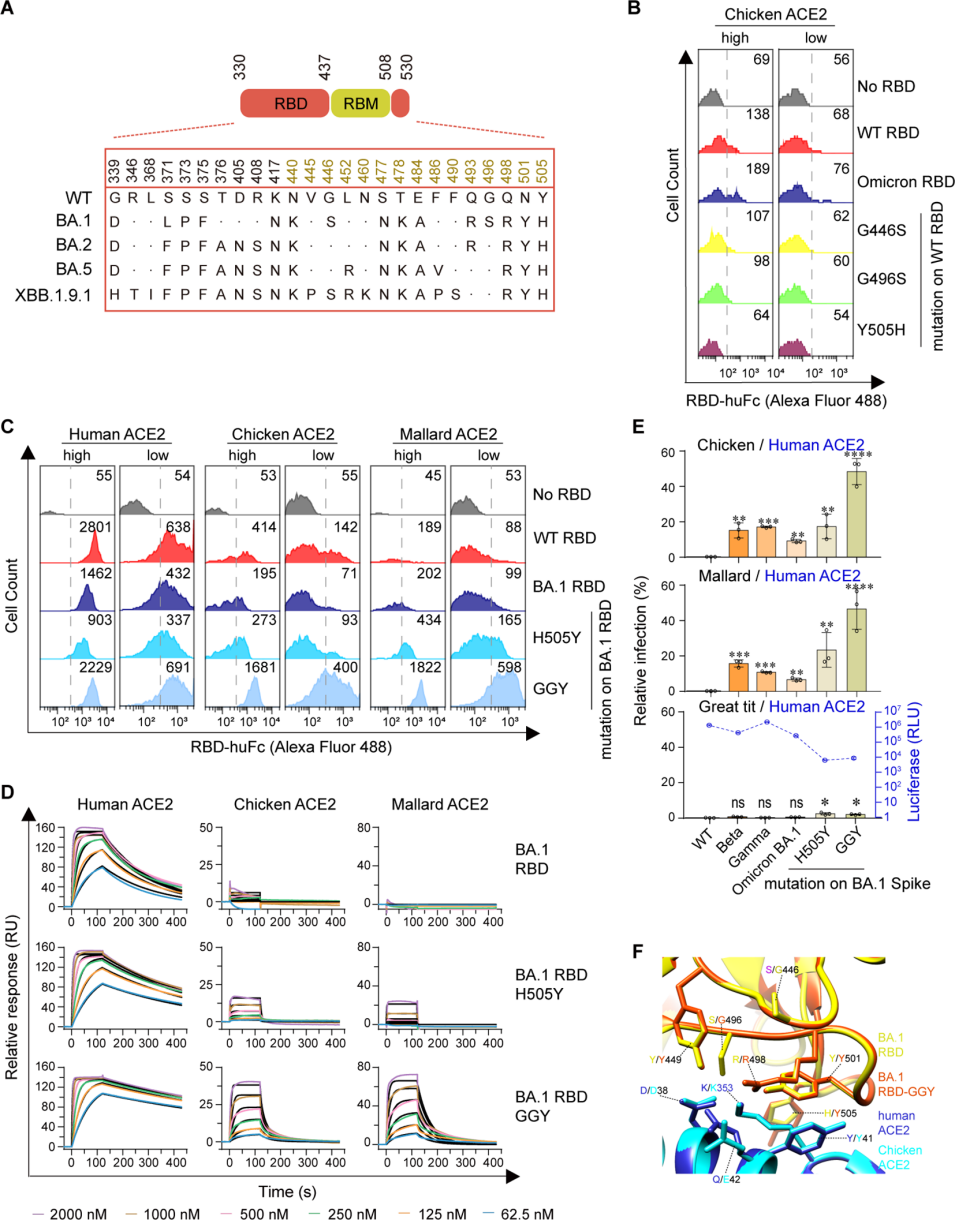
Omicron BA.1 RBD reversion mutations enable efficient Avian ACE2 binding. **(A)** The spike genes of SARS-CoV-2 WT and Omicron sublineages investigated in this study are illustrated. The RBD is highlighted in red, and the RBM is in goldenrod. **(B, C)** Flow cytometry detection of interactions between the indicated RBD proteins and cell surface-expressed human, chicken, or mallard ACE2 protein (GGY: S446G-S496G-H505Y). **(D)** SPR measurements of interaction kinetics were performed using dimeric human, chicken, or mallard ACE2 ectodomain as immobilized ligands and one of the indicated monomeric RBD proteins as analyte. The raw curves are presented in colors and the fitted curves are shown in black. **(E)** 293T cells expressing ACE2 were infected with the indicated SARS-CoV-2 spike-pseudotyped reporter viruses similar to **Figure 1E**. **(F)** A structural model of the chicken ACE2 protein was generated using the cryo-EM structure of the SARS-CoV-2 Omicron BA.1 spike protein in complex with human ACE2 (PDB accession no. 7T9L) as a modeling template. A structural model of the Omicron BA.1 RBD-GGY mutant was generated using the same structural template. Modeled chicken ACE2 structure and Omicron BA.1 RBD-GGY mutant structure were superimposed to the structure of Omicron BA.1 RBD in complex with human ACE2 (PDB accession no. 7T9L). Red and yellow indicate the Omicron BA.1 RBD and Omicron BA.1 RBD-GGY, respectively. Blue and cyan indicate human and chicken ACE2 proteins, respectively. Key residues involved in the high-affinity interaction between Omicron BA.1 RBD-GGY and chicken ACE2 were shown and labeled. ****, *P* < 0.001; ***, *P* < 0.001; **, *P* <0.01; *, *P* <0.05; n.s., not significant [*P* > 0.05].

Although part of the BA.1 RBD’s signature mutations (K417N-E484A-N501Y) resembled Beta RBD’s K417N-E484K-N501Y signature mutations (**Figure 3A**), BA.1 RBD barely bound to chicken or mallard ACE2 (**Figures 1**, **2**). To explore the potential mechanisms underlying the non-permissiveness of avian ACE2 to Omicron variants, the BA.1 RBD mutations were individually introduced into the WT strain. Flow cytometry assays showed that G446S, G496S, and Y505H adversely affected chicken ACE2–RBD interactions (**Figure 3B**; **Supplementary Figures S4A, B**). A triple reversal mutant GGY (S446G-S496G-H505Y) of the BA.1 RBD and a single reversal mutant H505Y were constructed. Cell surface staining and SPR assays demonstrated that the GGY mutations efficiently restored binding to chicken and mallard ACE2 (**Figures 3C, D**; **Supplementary Figures S4C-E**; **Supplementary Table S3**). Moreover, mutant BA.1 spike containing the GGY mutations was capable of utilizing chicken and mallard ACE2 for cell entry at approximately 50% efficiency compared to human ACE2 (**Figure 3E**). Combining the subsequent structural analyses (Mannar et al., 2022), a hydrogen bond interaction between RBD-Y449 and ACE2-D38, salt bridge interactions between RBD-R498 and ACE2-D38/E42, a perpendicular π–π stacking interaction between RBD-Y501 and ACE2-Y41, and sandwiched hydrophobic interactions among the aromatic ring of RBD-Y505 residue, the aliphatic part of ACE2-K353 residue, and the aromatic ring of ACE2-Y41 residue were annotated among the interface of RBD and ACE2 (Han et al., 2022) (**Figure 3F**). In summary, although avian ACE2 barely binds to Omicron BA.1 RBD, the reversal mutation of S446G-S496G-H505Y in BA.1 RBD restore efficient binding, resulting in virus entry mediated by chicken or mallard ACE2.

### Emerging Omicron sublineages utilize avian ACE2 for viral entry

**Figure 4:**
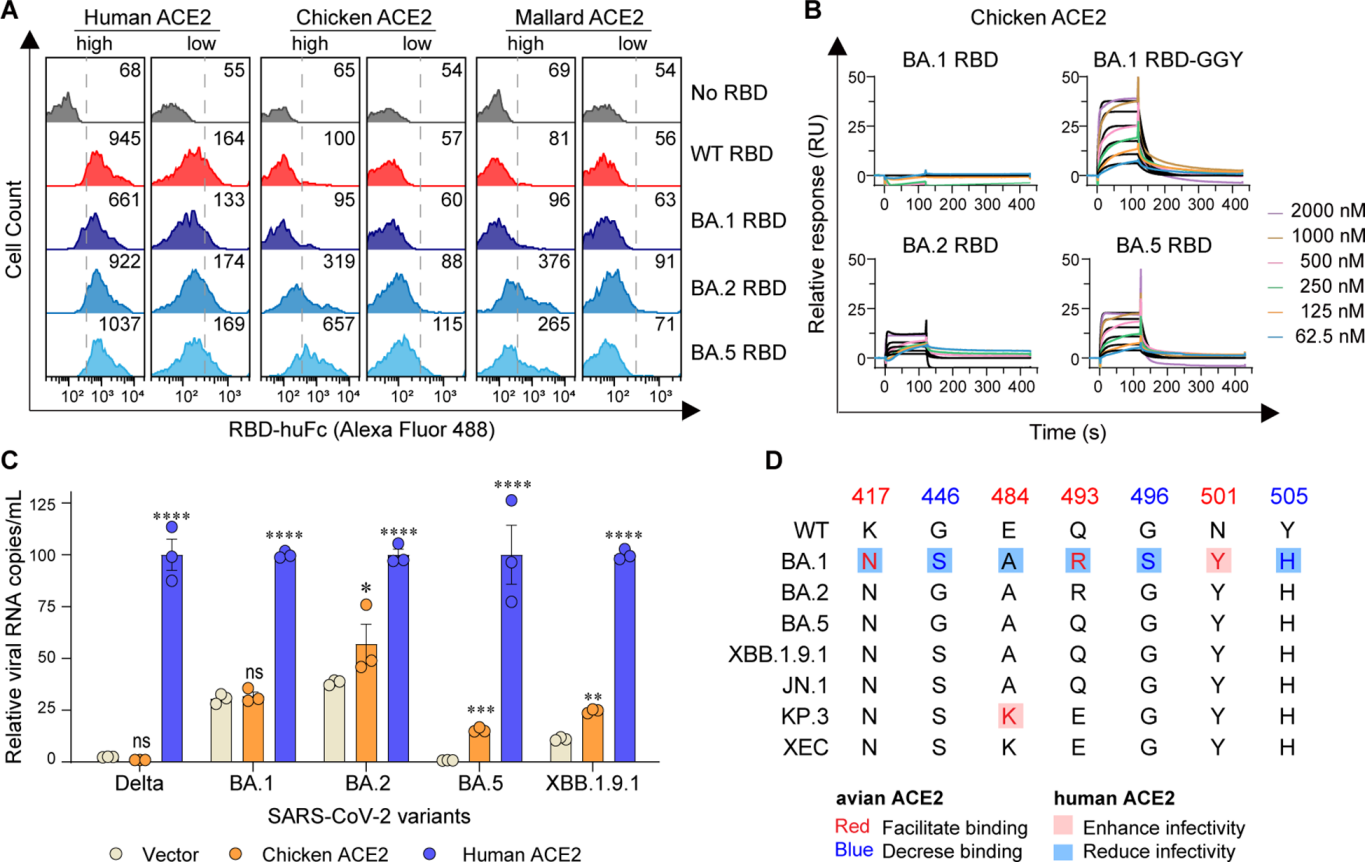
Emerging Omicron sublineages utilize avian ACE2 for viral entry. **(A)** Flow cytometry detection of interactions between the indicated RBD proteins and cell surface-expressed human, chicken, or mallard ACE2 proteins. **(B)** SPR measurements of interaction kinetics were performed using dimeric chicken ACE2 ectodomain as the immobilized ligand and one of the indicated monomeric RBD proteins as analytes. The raw curves are presented in colors and the fitted curves are shown in black. **(C)** HeLa cells expressing human or chicken ACE2 were infected with the indicated SARS-CoV-2 authentic virus at an MOI of 0.05 and harvested at 48 h post-infection (hpi) for viral RNA quantification. **(D)** The spike genes from SARS-CoV-2 WT and Omicron sublineages investigated in this study are illustrated. ****, *P* < 0.001; ***, *P* < 0.001; **, *P* <0.01; n.s., not significant [*P* > 0.05].

Omicron BA.1 is the earliest identified sublineage of the Omicron variant family and harbors numerous mutations in the spike protein, which significantly enhance transmissibility and immune evasion. The later-emerging Omicron sublineages BA.2 and BA.5 accumulated several additional mutations within the RBD, notably reversion mutations at positions 446 and 496 (**Figure 3A**). Cell surface staining assays showed that both BA.2 and BA.5 RBDs exhibited increased binding ability to human ACE2 and, importantly, markedly improved binding to chicken and mallard ACE2 compared to BA.1 RBD (**Figure 4A**; **Supplementary Figures S5A, B**). To quantify these interactions, we performed SPR analyses. The results showed that BA.5 gained the ability to bind chicken ACE2—containing the S446G and S496G reversal mutations—although with lower affinity than the BA.1 GGY mutant (**Figure 4B**; **Supplementary Table S4**). Consistent with RBD binding assays, HeLa cells expressing chicken ACE2 barely supported infection by the SARS-CoV-2 Delta variant or Omicron BA.1. In contrast, BA.2, BA.5, and XBB.1.9.1 were able to utilize chicken ACE2 to mediate efficient viral entry in HeLa cells (**Figure 4C**). Overall, these data indicate that Omicron sublineages (BA.2, BA.5, XBB) have progressively acquired mutations that enhance their ability to utilize avian ACE2 for cellular entry.

## Discussion

The potential cross-species transmission of SARS-CoV-2, which provides expanded reservoirs, may increase the uncertainty of future pandemics. Here, we found that WT SARS-CoV-2-encoded RBD failed to bind to chicken ACE2, while orthologous ACE2 of certain avian species were capable of binding to RBDs encoded by SARS-CoV-2 Alpha and Beta variants, along with the Gamma variant, resulting in the potential accessibility of these variants to avian species. The triple reversal mutant GGY (S446G, S496G, and H505Y) in Omicron BA.1 RBD restored its ability to bind avian ACE2 and behaved similarly to the Beta and Gamma variants. Further affinity assays and viral infections validated such reversal effects. By aligning the sequences of WT, BA.1, and the later sublineages BA.2, BA.4/5, XBB.1.9.1, and BQ.1.1, we observed that the S446G and S496G reversal mutations were already carried in their RBDs (Planas et al., 2023).

In addition, comparison of RBD diversity among newly emerged Omicron sublineages and all tested strains indicated four key residues were characterized as enhancing avian ACE2 binding, along with three other residues with negative effects (**Figure 4D**). Furthermore, these also implied that the preferences of all three recent sublineages (JN.1, KP.3, and XEC) exhibited the tendency toward avian species (**Figure 4D**). Notably, G446S, G496S, and Y505H significantly promote antibody evasion, though they also result in reduced binding affinity to hACE2 (Cao et al., 2022; Han et al., 2022; Liu et al., 2022; Yao et al., 2024). G496S is unique to Omicron BA.1 and has been confirmed to diminish binding affinity to hACE2 compared to the BA.2 sublineage (Han et al., 2022; Kimura et al., 2022; Li et al., 2022). Conversely, the E484K, Q493H, and N501Y mutations are consistently present across most strains and facilitate binding to both human (Sun et al., 2021; Yao et al., 2024) and avian orthologs. K417N and E484A reduce transmissibility among humans (Sun et al., 2021; McCallum et al., 2022; Yao et al., 2024), promote the avian ACE2 binding. Collectively, these mutagenesis studies indicated that the host range of these newly evolved SARS-CoV-2 variants is potentially expanded. Considering the limited boundary between humans and many avian species, the potential overcoming of host range barriers could lead to future cross-species pandemics, accompanied by higher mutation rates and the emergence of new variants.

However, no viral proteins were detected in the chicken-derived DF1 cell line, whether expressing human or chicken ACE2 (data not shown). In addition, in the presence of TMPRSS2 (transmembrane serine protease 2), which is preferentially utilized by SARS-CoV-2 to direct entry via fusion of the viral envelope to the cellular membrane (Koch et al., 2021), the pseudoviruses could not infect chicken cells (data not shown). Thus far, no official cases of *in vivo* avian infection by SARS-CoV-2 have been reported (Praharaj et al., 2021; Wang L. et al., 2021). Although the spike RBD is necessary for ACE2-mediated entry, this suggests that receptor binding alone may not be sufficient to enable productive infection in avian species. Specifically, based on the fact that alpha and betaCoVs are not found in birds while gamma and deltaCoVs are, this indicates that additional critical determinants beyond receptor engagement govern vertebrate host specificity. In addition to ACE2 usage, the AXL receptor could serve as a binding site for emerging SARS-CoV-2 variants (Lei et al., 2023). Beyond the entry step, downstream steps of viral replication—such as spike cleavage efficiency (by TMPRSS2, cathepsins, or furin), intracellular fusion machinery compatibility (with host co-factors), or host innate immune evasion (e.g., species-specific interferon-stimulated genes between avian and mammalian hosts)—serve as critical bottlenecks preventing beta-coronavirus replication in avian cells.

Notably, single-cell transcriptomic analyses revealed that genetic regulatory networks in lung cell atlases are deeply conserved among mammals, reptiles, and birds (Chen et al., 2021), suggesting a shared cellular framework that could theoretically support viral replication if entry barriers were overcome. The genetic diversity of beta-coronaviruses has maintained widespread transmission in recent years. Avian ACE2 orthologs exhibit high sequence consistency at the interface with SARS-CoV-2 RBD. The fact that SARS-CoV-2 RBD variants can bind avian ACE2 suggests a possible risk of cross-species transmission. Our results show that, beyond ACE2 binding, it is also important to assess the later steps of virus–host interaction when evaluating the risk of cross-species spread.

## Material and method

### Cells, plasmids, and viruses

Cells, plasmids, SARS-CoV-2 pseudoviruses, and SARS-CoV-2 strains were described in our previous studies (Li et al., 2020; Yao et al., 2023; Sun et al., 2024). 293T cells, Vero cells, and HeLa cells were maintained in Dulbecco’s Modified Eagle Medium (DMEM, Life Technologies) at 37°C in a 5% CO2-humidified incubator. Growth medium was supplemented with 2 mM Glutamax-I (Gibco, Cat. No. 35050061), 100 µM non-essential amino acids (Gibco, Cat. No. 11140050), 100 U/mL penicillin, 100 µg/mL streptomycin (Gibco, Cat. No. 15140122), and 10% heat-inactivated FBS (Gibco, Cat. No. 10099141C). 293F cells were maintained in SMM 293-TII serum-free medium (Sino Biological, Cat. No. M293TII) at 37°C, 8% CO2, in a shaker incubator at 125 rpm.

Plasmids expressing spike variants, S-tagged ACE2, soluble ACE2 (residues 18–740), dimeric RBD, and monomeric RBD were described in our previous studies (Li et al., 2020; Yao et al., 2023). Briefly, DNA fragments encoding N-terminally S-tagged ACE2 orthologs were cloned into the pQCXIP plasmid (Clontech). Plasmids encoding soluble ACE2 orthologs (18-740aa) and dimeric RBD variants were generated by cloning each gene fragment into a pCAGGS-based human IgG1 Fc fusion expression plasmid. Plasmids for monomeric RBD with an additional C-terminal HRV 3C protease cleavage site were cloned into the same Fc fusion vector.

MLV retroviral vector-based SARS-CoV-2 spike pseudotypes were produced in 293T cells and titrated using a reverse transcriptase activity assay (Yao et al., 2023). In brief, 293T cells were transfected with plasmids encoding a spike variant, murine leukemia virus (MLV) Gag and Pol proteins, and a pQCXIP-based luciferase reporter plasmid. Cell culture supernatants were collected 48 h post-transfection. SARS-CoV-2 strains were described in our previous study and were passaged on Vero E6 cells (Sun et al., 2024). SARS-CoV-2 titers were determined by plaque assays in Vero E6 cells.

### Production and purification of ACE2 or RBD proteins

Protein production was performed as described previously (Yao et al., 2023). 293F cells were transfected with ACE2 or RBD expression plasmids. IgG Fc-containing fusion proteins were purified from cell culture supernatants using Protein A Sepharose CL-4B (GE Healthcare, Cat. No. 17-0780-01), eluted with 0.1 M citric acid (pH 4.5), and neutralized with 1 M Tris-HCl (pH 9.0). RBD-Fc proteins containing an HRV 3C protease cleavage site were cleaved in-column using His-tagged HRV 3C protease (Thermo, Cat. No. 88946) to release RBD monomers. HRV 3C protease was then removed using HisPur Ni-NTA Resin (Thermo, Cat. No. 88221).

### Flow cytometry for detecting interactions of RBD and ACE2 orthologs

293T cells in each well of a 12-well plate were transfected with 0.4 µL of Lipofectamine 2000 (Life Technologies, Cat. No. 11668019) and 125 ng of a plasmid encoding an ACE2 ortholog or its mutant. At 36 h post-transfection, cells were detached with 5 mM EDTA (Life Technologies, Cat. No. 15575020). Cells were stained with 2 µg/mL rabbit anti-S-tag IgG polyclonal antibody (Abcam, Cat. No. ab183674) at 37°C for 30 min, washed with PBS, and incubated with 5 µg/mL RBD-huFc proteins at 37°C for 15 min. Then, cells were stained with 2 µg/mL Alexa488-conjugated goat anti-human IgG secondary antibody (Invitrogen, Cat. No. A11013) and Alexa568-conjugated goat anti-rabbit IgG (Invitrogen, Cat. No. A11011) at room temperature for 20 min. After washing with PBS, cells were fixed with 0.1% paraformaldehyde in PBS and analyzed using an Attune NxT flow cytometer (Thermo Fisher). Data were collected using Attune NxT Software v 4.2. For each sample, 2,400 ACE2 low-gated cell events were analyzed.

### Surface plasmon resonance assay

SPR assays were performed at 25°C using the Biacore 8K High-throughput Intermolecular Interaction Analysis System, following the manufacturer’s instructions. ACE2-huFc constructs were diluted to 10 µg/mL in 1× assay buffer containing 150 mM NaCl, 0.05% Tween-20, and 10 mM HEPES (pH 7.4). ACE2-huFc was captured on a Protein A sensor chip (GE Healthcare) to a level of 300–700 response units (RUs). RBD monomers were serially diluted to 2000 nM, 1000 nM, 500 nM, 250 nM, 125 nM and 62.5 nM. Each binding cycle included 45 s capture, 120 s contact, 300 s dissociation, and 120 s regeneration. Binding data were fitted to a 1:1 binding model using Biacore Evaluation Software.

### SARS-CoV-2 infection assay

The pseudovirus infection assay was performed as described previously (Yao et al., 2023). 293T cells were reverse transfected with 0.15 µL of Lipofectamine 2000 (Life Technologies, Cat. No. 11668019) and 60 ng of a vector control or a plasmid encoding an ACE2 ortholog or its mutant. At 24 h post-transfection, cells were infected with SARS-CoV-2 pseudoviral particles equivalent to 8×10^10^ U reverse transcriptase. Cell culture supernatants were collected at 48 h post-infection and subjected to a Gaussia luciferase assay using a Centro LB 960 microplate luminometer (Berthold Technologies). S-tagged ACE2 expression in 293T cells was detected using Western blot at 24 h post-transfection using a mouse anti-S-tag monoclonal antibody 6.2 (Invitrogen, Cat. No. MA1-981) and an HRP-conjugated goat anti-mouse IgG Fc antibody (Invitrogen, Cat. No. 31437). Mouse anti beta-actin IgG monoclonal antibody BA3R (Invitrogen, Cat. MA5-15739) was used as a loading control.

Authentic virus infection assays were performed as described previously (Sun et al., 2024). HeLa cells were reverse transfected with 60 ng plasmid encoding an ACE2 ortholog or its mutant. Twenty-four hours post transfection, cells in each well were infected with the authentic SARS-CoV-2 virus with MOI = 0.05. Cell culture supernatants were collected, and viral RNA was extracted. RT-qPCR was performed using the One Step PrimeScript RT-PCR Kit (TAKARA, RR064A) on a CFX Connect Real‐Time system (Bio‐Rad, CA, USA). The primers and probes for quantitative PCR were ORF1ab-qF: 5′-CCCTGTGGGTTTTACACTTAA-3′, ORF1ab-qR: 5′-AC GATTGTGCATCAGCTGA-3′, and probe: 5′-FAM-CCGTCTGCGGTATGTGGAAAGG TTATGG-3′-BHQ1.

### Experimental data collection and statistical analysis

Data shown in the figures are representative of two or three independent experiments, and data points represent mean values ± s.d of three biological replicates. Significance values were calculated with one-way analysis of variance (ANOVA) and Dunnett’s multiple-comparison test (****, *P* < 0.001; ***, *P* < 0.001; **, *P* <0.01; *, *P* <0.05; n.s., not significant [*P* > 0.05]). Flow cytometry data were analyzed by FlowJo V10 software. The SPR data were fitted to a 1:1 binding model using Biacore Evaluation Software. Image Lab software (Bio-Rad) was used to collect SDS-PAGE and Western blot image data. MikroWin 2000 software (Berthold Technologies) was used to collect luciferase assay data. ACE2 sequence alignment was performed with BioEdit Sequence Alignment Editor. Structural models of animal ACE2 proteins were generated using the SWISS-MODEL protein structure homology-modeling server. GraphPad Prism 9.0 software was used for the preparation of bar graphs and statistical analyses. Adobe Illustrator 2022 was used for the preparation of the figures for the manuscript.

## Data Availability

The datasets presented in this study can be found in online repositories. The names of the repository/repositories and accession number(s) can be found in the article/**Supplementary Material**.
