# Supplementary material for: Characterization of key spike RBD residues influencing SARS-CoV-2 variant adaptation to avian ACE2
Source: Front Cell Infect Microbiol. 2025 Jul 29;15:1631926. doi: 10.3389/fcimb.2025.1631926 (PMC12339479; doi:10.3389/fcimb.2025.1631926)
Supplement: Supplementary file 1 [file DataSheet1.pdf]

Supporting Information for

Characterization of Key Spike RBD Residues Influencing SARS-CoV-2 Variant Adaptation to Avian ACE2

Weitong Yao[1]†, Yujun Li[2]†*, Huize Sun[1], Danting Ma[3], Xiaojuan Tang[4,5], Aiping Zeng[6]*, Fang Huang[1]*

Affiliations:

[1] Hubei JiangXia Laboratory, Wuhan, Hubei 430200, China

[2] Biosafety Level 3 Laboratory of Shenzhen University, Shenzhen, Guangdong 518000, China

[3] NHC Key Laboratory of Hormones and Development, Tianjin Key Laboratory of Metabolic Diseases, Chu Hsien-I Memorial Hospital & Tianjin Institute of Endocrinology, Tianjin Medical University , Tianjin 300134, China

[4] School of Chemical Biology and Biotechnology, Peking University Shenzhen Graduate School, Shenzhen, Guangdong 518055, China

[5] Shenzhen Bay Laboratory, Shenzhen, Guangdong 518132, China

[6] Department of Ophthalmology, Union Hospital, Tongji Medical College, Huazhong University of Science and Technology, Wuhan, Hubei 430200, China

† Equal contribution

* Correspondence to huangf@wh.iov.cn; liyujun@szu.edu.cn (Y.L.), aiping_zeng@163.com

This PDF file includes:

Table S1 to S4

Legends for Figures S1 to S5

S1 to S5 Figs

Table S1. SPR data for the interaction of Chicken ACE2 with SARS-CoV-2 RBDs

Loading Sample (ACE2-hFc)	Analytes (RBD-monomer)	Rmax (RU)	ka ($\times 10^5$ M-1 s-1)	kd ($\times 10^{-3}$ s-1)	KD (nM)
Chicken	WT	693.2	N.D.	N.D.	N.D.
Chicken	Alpha	2089.6	0.003	124	409000
Chicken	Beta	23219.2	0.002	304	1740000
Chicken	Gamma	747.8	0.042	189	44700
Chicken	Delta	95.4	0.092	240	26000
Chicken	Omicron	9.6	N.D.	N.D.	N.D.

Table S2. Accession numbers of avian ACE2s

No.	Species name used in the text	Binomial name	NCBI Reference Sequence ID or Genebank ID
1	Tufted duck	Aythya fuligula	XP_032058386
2	Northern mallard	Anas platyrhynchos platyrhynchos	U3J4G2
3	Magpie goose	Anseranas semipalmata	NXI70543
4	Anser	Anser cygnoides domesticus	XP_013039300
5	Turkey	Meleagris gallopavo	XP_019467554
6	Australian brush turkey	Alectura lathami	NXL86717
7	Helmeted guineafowl	Numida meleagris	XP_021240731
8	Japanese quail	Coturnix japonica	XP_015742063
9	Marbled wood quail	Odontophorus gujanensis	NXJ08219
10	Mariana crow	Corvus kubaryi	XP_041893393
11	Eurasian tree sparrow	Passer montanus	XP_039570234
12	Chipping sparrow	Spizella passerina	NXX65640
13	Kakapo	Strigops habroptila	A0A672V5V3
14	Blackcap	Sylvia atricapilla	NWY36770
15	Ardeotis	Ardeotis kori	NXE27194
16	Mallard	Anas platyrhynchos	XP_012949915
17	Great Tit	Parus major	XP_015486815
18	Great cormorant	Phalacrocorax carbo	XP_009509070.1
19	Little egret	Egretta garzetta	XP_009638257.1
20	Boat-billed heron	Cochlearius cochlearius	NXE75379.1
21	Eurasian golden oriole	Oriolus oriolus	NXO17556.1
22	Red-winged blackbird	Agelaius phoeniceus	A0A7K7JMF2
23	Bald eagle	Haliaeetus leucocephalus	XP_010579828.1
24	White-tailed eagle	Haliaeetus albicilla	XP_009925641.1
25	Lesser kestrel	Falco naumanni	XP_040438616.1
26	Barn owl	Tyto alba	XP_042644640.1
27	Burrowing owl	Athene cunicularia	XP_026705725.1
28	Chuck-will's-widow	Antrostomus carolinensis	XP_010169238.1
29	Penelope	Penelope pileata	NXC47207.1
30	Dalmatian pelican	Pelecanus crispus	XP_009478920.1

Table S3. SPR data for the interactions between animal ACE2 orthologs and Omicron RBD mutants

Loading Sample (ACE2-hFc)	Analytes (RBD-monomer)	Rmax (RU)	ka ($\times 10^5$ M-1 s-1)	kd ($\times 10^{-3}$ s-1)	KD (nM)
Human	BA.1	154.3	1.6	5.25	32.7
Human	BA.1-H505Y	148.0	1.43	2.27	15.9
Human	BA.1-GGY	137.1	2.37	1.04	4.42
Chicken	BA.1	10.7	N.D.	N.D.	N.D.
Chicken	BA.1-H505Y	27.3	1.32	186	1420
Chicken	BA.1-GGY	48.8	1120	616	551
Mallard	BA.1	N.D.	N.D.	N.D.	N.D.
Mallard	BA.1-H505Y	179.3	1.01	1510	15000
Mallard	BA.1-GGY	77.1	0.622	20.4	328

Table S4. SPR data for the interactions between Chicken ACE and Omicron RBD variants

Loading Sample (ACE2-hFc)	Analytes (RBD-monomer)	Rmax (RU)	ka ($\times 10^5$ M-1 s-1)	kd ($\times 10^{-3}$ s-1)	KD (nM)
Chicken	BA.1	N.D.	N.D.	N.D.	N.D.
Chicken	BA.1-GGY	45	0.989	39	394
Chicken	BA.2	13.2	2.58	86.1	334
Chicken	BA.5	27.1	1.50	56.1	375

Figure Legends

Figure S1. Alpha, Beta, and Gamma variants can bind avian ACE2 and utilize it for entry. (A, C) Dot plot raw data for the flow cytometry data shown in Figures 1A, 1D. (B) SDS-PAGE images of purified human IgG1 Fc fusion proteins of the indicated avian ACE2 (ACE2-huFc) used in Figure 1B. (D) 293T cells transfected with the indicated ACE2 genes (S-tagged) were collected at 24 hours post transfection. Expression levels of the ACE2 proteins were detected using Western Blot with an anti-S-tag antibody.

Figure S2. Seqence alignment of avian ACE2. Amino acid sequences of the indicated avian ACE2 orthologs are aligned with human ACE2 and only residues potentially interact with SARS-CoV-2 RBD are shown here. The numbering is based on human ACE2 protein. The residues different from the corresponding ones in chicken ACE2 are highlighted in blue. Accession numbers of the indicated avian ACE2 orthologs are shown in Table S2.

Figure S3. Avian ACE2 orthologs can bind SARS-CoV-2 variants RBDs. (A-B) Dot plot raw data for the flow cytometry data shown in Figures 2A, 2B. (C-D) 293T cells expressing ACE2 were infected with the WT, and Alpha SARS-CoV-2 spike-pseudotyped reporter viruses. Infection signals of each pseudovirus supported by indicated ACE2 proteins were calculated as percentage of infection relative to infection signals of corresponding pseudovirus supported by human ACE2 (EEN: T27E-A30E-D38N, TAD: E27T-E30A-N38D). (E) 293T cells transfected with the indicated ACE2 genes (S-tagged) were collected at 24 hours post transfection. Expression levels of the ACE2 proteins were detected using Western Blot with an anti-S-tag antibody.

Figure S4. Omicron gain strong binding to chicken ACE2 after three reversal mutations. (A, B) Flow cytometry detection of interactions between the indicated RBD dimers and cell surface expressed chicken ACE2 protein. (C) Dot plot raw data for the flow cytometry data shown in Figure 3C. (D-E) SDS-PAGE images of purified RBD-huFc proteins and monomeric RBD proteins used in Figures 3C-D.

Figure S5. Emerging Omicron sublineages utilize avian ACE2 for viral entry. (A) Dot plot raw data for the flow cytometry data shown in Figure 4A. (B-C) SDS-PAGE images of purified RBD-huFc proteins and monomeric RBD proteins used in Figures 4A-B.

Fig S1

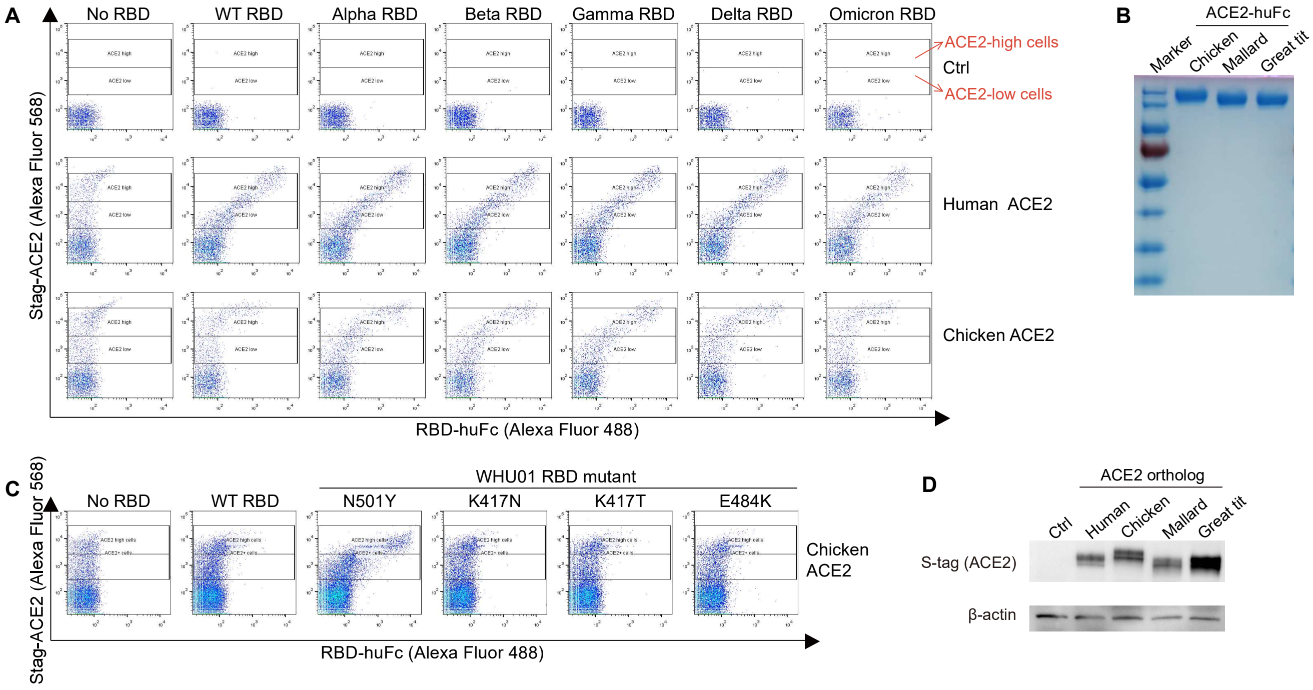
A
No RBD
WT RBD
Alpha RBD
Beta RBD
Gamma RBD
Delta RBD
Omicron RBD
ACE2-high cells
Ctrl
ACE2-low cells
Human ACE2
Chicken ACE2
Stag-ACE2 (Alexa Fluor 568)
RBD-huFc (Alexa Fluor 488)
B
ACE2-huFc
Marker
Chicken
Mallard
Great tit
C
WHU01 RBD mutant
No RBD
WT RBD
N501Y
K417N
K417T
E484K
Chicken ACE2
Stag-ACE2 (Alexa Fluor 568)
RBD-huFc (Alexa Fluor 488)
D
ACE2 ortholog
Ctrl
Human
Chicken
Mallard
Great tit
S-tag (ACE2)
β-actin

Fig S2

	19	24	27	28	30	31	34	35	37	38	41	42	45	79	82	83	330	353	354	355	357	393
human	S	Q	T	F	D	K	H	E	E	D	Y	Q	L	L	M	Y	N	K	G	D	R	R
**Gallus gallus (chicken)**	D	E	T	F	A	E	V	R	E	D	Y	E	L	N	R	F	N	K	N	D	R	R
Meleagris gallopavo (turkey)	D	E	T	F	A	E	V	R	E	D	Y	E	L	N	R	F	N	K	N	D	R	R
Numida meleagris (helmeted guineafowl)	D	E	I	F	A	E	V	R	E	D	Y	E	L	N	R	F	N	K	N	D	R	R
Coturnix japonica (Japanese quail)	D	E	K	F	A	E	V	R	E	D	Y	E	L	N	R	F	N	K	N	D	R	R
Alectura lathami (Australian brush turkey)	D	Q	T	F	A	E	V	V	E	D	Y	E	L	N	R	F	N	K	N	D	R	R
Penelope pileata	D	E	M	F	A	E	V	R	E	D	Y	E	L	N	R	F	N	K	G	D	R	R
**Anas platyrhynchos (mallard)**	D	Q	M	F	A	E	V	R	E	D	Y	E	L	N	N	F	N	K	N	D	R	R
Anas platyrhynchos platyrhynchos (Northern mallard)	D	Q	M	F	A	E	V	R	E	D	Y	E	L	N	N	F	N	K	N	D	R	R
Aythya fuligula (Tufted duck) (Anas fuligula)	D	Q	M	F	A	E	V	R	E	D	Y	E	L	N	N	F	N	K	N	D	R	R
Anseranas semipalmata (Magpie goose) (Anas semipalmata)	D	Q	M	F	A	E	V	R	E	D	Y	E	L	N	S	F	N	K	N	D	R	R
Anser cygnoides domesticus	D	Q	M	F	A	E	V	R	E	D	Y	E	L	N	S	F	N	K	D	D	R	R
Tyto alba (Barn owl)	D	Q	T	F	E	E	R	R	E	D	Y	E	L	N	R	F	N	K	N	D	R	R
Athene cunicularia (burrowing owl)	D	Q	M	F	E	E	R	R	E	D	Y	E	L	N	S	F	N	K	N	D	R	R
Antrostomus carolinensis (chuck-will's-widow)	D	Q	I	F	E	E	R	R	E	D	Y	E	L	-	S	F	N	K	N	D	R	R
Phalacrocorax carbo (great cormorant)	D	Q	T	F	E	E	R	R	E	N	Y	E	L	N	R	F	N	K	K	D	R	R
Corvus kubaryi (Mariana crow)	D	Q	M	F	E	E	R	R	E	N	Y	E	L	N	S	F	N	K	N	D	R	R
Strigops habroptila (Kakapo)	D	Q	M	F	E	E	R	R	E	N	Y	E	L	N	T	F	N	K	N	D	R	R
Haliaeetus albicilla (white-tailed eagle)	D	Q	M	F	E	E	R	R	E	N	Y	E	L	-	S	F	N	K	N	D	R	R
Ardeotis kori	D	Q	V	F	E	E	R	R	E	N	Y	E	L	N	S	F	N	K	N	D	R	R
Falco naumanni (lesser kestrel)	D	E	M	F	E	E	R	R	E	N	Y	E	L	N	S	F	N	K	N	D	R	R
Haliaeetus leucocephalus (bald eagle)	-	-	M	F	E	E	R	R	E	N	Y	E	L	N	S	F	N	K	N	D	R	R
Cochlearius cochlearius (boat-billed heron)	D	Q	R	F	E	E	R	R	E	N	Y	E	L	N	S	F	N	K	N	D	R	R
Pelecanus crispus (Dalmatian pelican)	N	Q	M	F	E	E	R	R	E	D	Y	E	L	N	S	F	N	K	N	D	R	R
Egretta garzetta (little egret)	N	Q	R	F	E	E	R	R	E	D	Y	E	L	K	-	-	N	K	N	D	R	R
Passer montanus (Eurasian tree sparrow)	D	Q	M	F	E	E	R	R	E	N	Y	E	I	N	K	F	N	K	N	D	R	R
Agelaius phoeniceus (Red-winged blackbird) (Oriolus phoeniceus)	D	Q	R	F	E	E	R	R	E	D	Y	E	I	N	K	F	N	K	N	D	R	R
Spizella passerina (Chipping sparrow)	S	Q	T	F	E	E	R	R	E	D	Y	E	I	N	K	F	N	K	N	D	R	R
Oriolus oriolus (Eurasian golden oriole) (Coracias oriolus)	N	Q	M	L	E	E	R	K	E	D	Y	N	I	Y	S	F	N	K	N	D	R	R
Odontophorus gujanensis (marbled wood quail)	D	E	I	F	A	E	T	R	E	D	Y	E	L	N	R	F	K	K	N	D	R	R
Sylvia atricapilla (blackcap)	D	Q	K	F	E	E	S	R	E	D	Y	E	I	N	S	F	N	K	G	D	R	R
**Parus major (Great Tit)**	N	E	E	F	E	E	M	R	E	N	Y	E	I	N	N	F	N	K	N	D	R	R

Fig S3

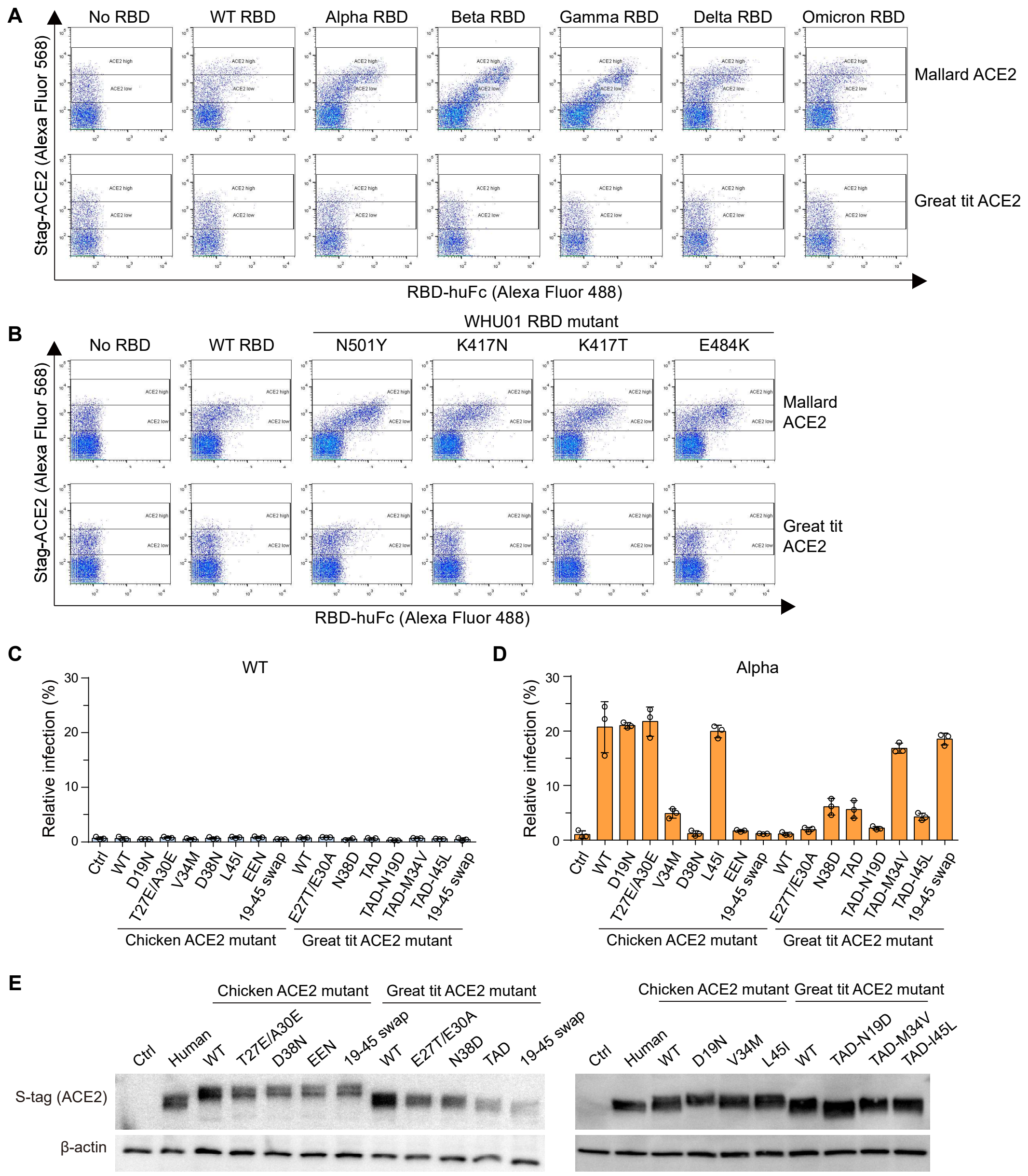
A
No RBD
WT RBD
Alpha RBD
Beta RBD
Gamma RBD
Delta RBD
Omicron RBD
Mallard ACE2
Great tit ACE2
Stag-ACE2 (Alexa Fluor 568)
RBD-huFc (Alexa Fluor 488)
B
WHU01 RBD mutant
No RBD
WT RBD
N501Y
K417N
K417T
E484K
Mallard ACE2
Great tit ACE2
Stag-ACE2 (Alexa Fluor 568)
RBD-huFc (Alexa Fluor 488)
C
WT
Relative infection (%)
Ctrl
WT
D19N
T27E/A30E
V34M
D38N
L45I
EEN
19-45 swap
WT
E27T/E30A
N38D
TAD
TAD-N19D
TAD-M34V
TAD-I45L
19-45 swap
Chicken ACE2 mutant
Great tit ACE2 mutant
D
Alpha
Relative infection (%)
Chicken ACE2 mutant
Great tit ACE2 mutant
E
Chicken ACE2 mutant
Great tit ACE2 mutant
Ctrl
Human
WT
T27E/A30E
D38N
EEN
19-45 swap
WT
E27T/E30A
N38D
TAD
19-45 swap
Ctrl
Human
WT
D19N
V34M
L45I
WT
TAD-N19D
TAD-M34V
TAD-I45L
S-tag (ACE2)
β-actin

Fig S4

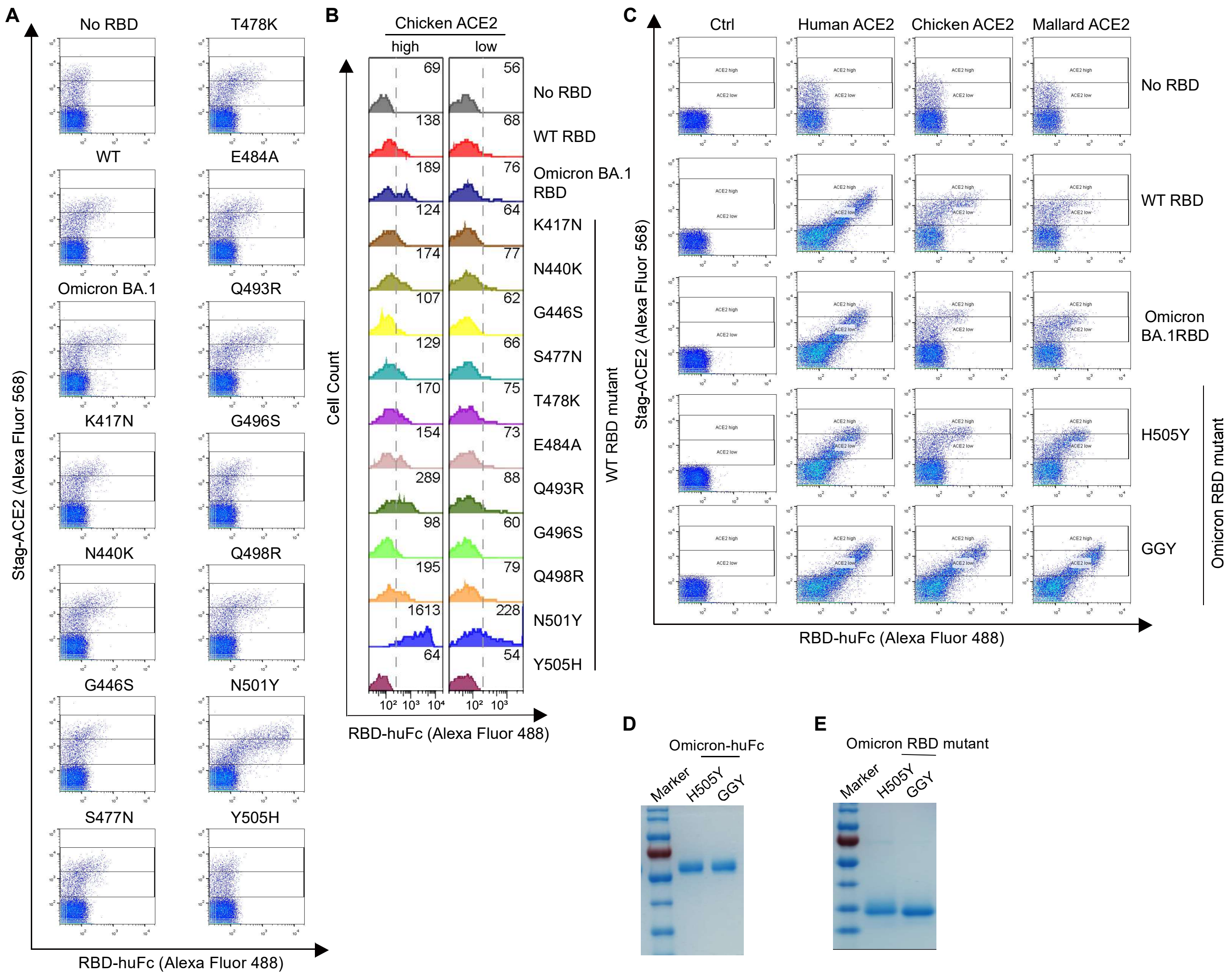
A
No RBD
T478K
WT
E484A
Omicron BA.1
Q493R
K417N
G496S
N440K
Q498R
G446S
N501Y
S477N
Y505H
Stag-ACE2 (Alexa Fluor 568)
RBD-huFc (Alexa Fluor 488)
B
Chicken ACE2
high
low
Cell Count
69
56
No RBD
138
68
WT RBD
189
76
Omicron BA.1 RBD
124
64
K417N
174
77
N440K
107
62
G446S
129
66
S477N
170
75
T478K
154
73
E484A
289
88
Q493R
98
60
G496S
195
79
Q498R
1613
228
N501Y
64
54
Y505H
WT RBD mutant
10^2 10^3 10^4
10^2 10^3
RBD-huFc (Alexa Fluor 488)
C
Ctrl
Human ACE2
Chicken ACE2
Mallard ACE2
No RBD
WT RBD
Omicron BA.1RBD
H505Y
GGY
Omicron RBD mutant
Stag-ACE2 (Alexa Fluor 568)
RBD-huFc (Alexa Fluor 488)
D
Omicron-huFc
Marker
H505Y
GGY
E
Omicron RBD mutant
Marker
H505Y
GGY

Fig S5

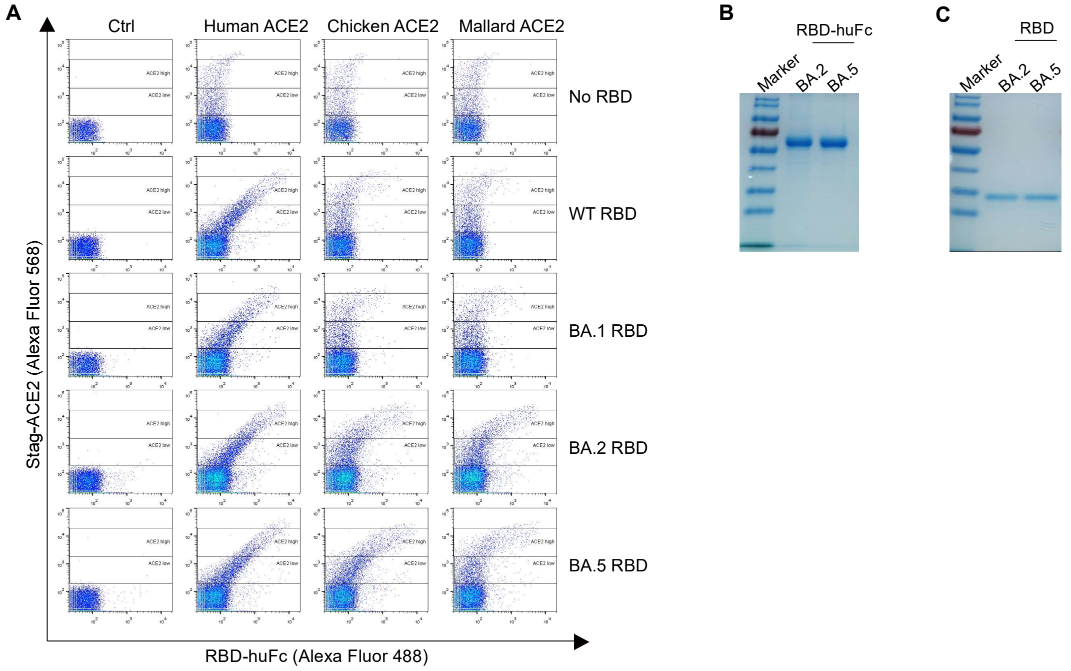

A
Ctrl
Human ACE2
Chicken ACE2
Mallard ACE2
No RBD
WT RBD
BA.1 RBD
BA.2 RBD
BA.5 RBD
Stag-ACE2 (Alexa Fluor 568)
RBD-huFc (Alexa Fluor 488)
B
RBD-huFc
Marker
BA.2
BA.5
C
RBD
Marker
BA.2
BA.5
